# D-dimer, Troponin, and Urea Level at Presentation With COVID-19 can Predict ICU Admission: A Single Centered Study

**DOI:** 10.3389/fmed.2020.585003

**Published:** 2020-12-09

**Authors:** Mahmood Y. Hachim, Ibrahim Y. Hachim, Kashif Bin Naeem, Haifa Hannawi, Issa Al Salmi, Suad Hannawi

**Affiliations:** ^1^College of Medicine, Mohammed bin Rashid University of Medicine and Health Sciences, Dubai, United Arab Emirates; ^2^Clinical Sciences Department, College of Medicine, University of Sharjah, Sharjah, United Arab Emirates; ^3^Ministry of Health and Prevention (MOHAP), Dubai, United Arab Emirates; ^4^The Royal Hospital, Muscat, Oman

**Keywords:** COVID-19, severe COVID-19 prediction, risk stratification, ICU - Intensive care unit, SARS-CoV-2

## Abstract

**Background:** Identifying clinical-features or a scoring-system to predict a benefit from hospital admission for patients with COVID-19 can be of great value for the decision-makers in the health sector. We aimed to identify differences in patients' demographic, clinical, laboratory, and radiological findings of COVID-19 positive cases to develop and validate a diagnostic-model predicting who will develop severe-form and who will need critical-care in the future.

**Methods:** In this observational retrospective study, COVID-19 positive cases (total 417) diagnosed in Al Kuwait Hospital, Dubai, UAE were recruited, and their prognosis in terms of admission to the hospital and the need for intensive care was reviewed until their tests turned negative. Patients were classified according to their clinical state into mild, moderate, severe, and critical. We retrieved all the baseline clinical data, laboratory, and radiological results and used them to identify parameters that can predict admission to the intensive care unit (ICU).

**Results:** Patients with ICU admission showed a distinct clinical, demographic as well as laboratory features when compared to patients who did not need ICU admission. This includes the elder age group, male gender, and presence of comorbidities like diabetes and history of hypertension. ROC and Precision-Recall curves showed that among all variables, D dimers (>1.5 mg/dl), Urea (>6.5 mmol/L), and Troponin (>13.5 ng/ml) could positively predict the admission to ICU in patients with COVID-19. On the other hand, decreased Lymphocyte count and albumin can predict admission to ICU in patients with COVID-19 with acceptable sensitivity (59.32, 95% CI [49.89–68.27]) and specificity (79.31, 95% CI [72.53–85.07]).

**Conclusion:** Using these three predictors with their cut of values can identify patients who are at risk of developing critical COVID-19 and might need aggressive intervention earlier in the course of the disease.

## Background

The pandemic of (COVID-19), which began at the end of 2019, represents an international public health emergency (1). Most patients with this disease suffer from mild to moderate illnesses (2). However, a small percentage of those patients suffer from more severe illnesses that can rapidly progress into a more critical form. This includes ARDS and acute respiratory failure, in addition to metabolic acidosis, coagulopathies, and septic shock (3). Depending on patient characteristics and the studied population, ICU admission varies between 5 and 16% of the total number of patients (4). The widespread of the disease led to a rapid overwhelming of the public health system of different countries, including the intensive care units (5), with some countries reaching a critical care crisis (6).

The current pandemic increased the burden substantially on acute and critical care services exceeding existing hospital capacity around the world (7). Managing the expected surges in intensive care capacity requires focused intensive care abilities and requirements to minimize loss of life and maintain control (8). Due to the increasing numbers of recorded positive COVID-19 cases, the medical teams in the front line are in urgent need of a tool that helps their clinical judgment to identify the few that will progress to critical cases.

A stratification tool for non-severe COVID-19 patients at admission can direct the resources and control the spread more efficiently and persevere the health team's power (9). The use of prediction models for COVID-19 will support medical decision making but are still poorly reported (10). The decisions of easing the preventive measures in countries that passed the peak of transmission are complicated with the anticipation of a second wave that necessitates a sufficiently prepared action plan for handling cases on admission (11). Identifying clinical features or a scoring system to predict a benefit from hospital admission for patients with COVID-19 can be of great value for the decision-makers in the health sector.

Right now, there are few reliable, applicable, or useable clinical models or scoring systems to predict if a tested positive for COVID-19 should be admitted to the hospital or asked to stay home especially in regions like the middle east using local patients parameters for prediction (12). During the manuscript writing, a scoring system of COVID-19 (CSS) was suggested that could help clinicians to identify high-risk patients with poor prognosis (13). Another promising predictive tool PREDI-CO score was suggested to be useful in resource allocation and treatment prioritization during the COVID-19 pandemic (14). We aimed to identify differences in patients' demographic, clinical, laboratory, and radiological findings between mild, severe, and critical cases of COVID-19 positive cases to develop and validate a diagnostic model predicting who will develop severe form and who will need critical care throughout the course of the disease.

## Materials and Methods

### Patients Data Collection

In this observational retrospective study, COVID-19 positive patients (total 417) admitted between January to June 2020 were recruited from Al Kuwait Hospital, Dubai, UAE. Those patients are consecutive patients approaching the hospital for COVID-19 related symptoms and were enrolled with the following inclusion criteria: Adult patients (above 18 years) with COVID-19 (confirmed by nasopharyngeal polymerase chain reaction; PCR positive sample). Complete current and past medical history, along with their demographic data, a history of a recent travel or contact with a confirmed or suspected case were documented.

The cohort was divided into two subgroups, the training group to identify predictors of ICU admission (*n* = 128) who were admitted between January and February and the validation group (*n* = 289) who were admitted in March till June 2020. The study was approved by the Ministry of Health and Prevention (MOHAP) Research Ethics Committee number (MOHAP/DXB-REC/MMM/NO.44/2020). The main presenting symptoms were enlisted, including [fever, cough, fatigue, anorexia, shortness of breath (SOB), sputum production, myalgias, headache, confusion, rhinorrhea, sore throat, hemoptysis, vomiting, diarrhea, nausea, anosmia, and ageusia]. Risk factors for severe illness were examined, including old age, cardiovascular diseases (CVD), diabetes mellitus (DM), hypertension (HTN), prior stroke and or transient ischemic attack, cancer, chronic lung disease, and chronic kidney disease (CKD).

### Patients Classification

Patients were classified according to “Clinical Management of Critically Ill COVID-19 Patients” guidelines (Version 1- April 15, 2020) issued by MOHAP (6). Accordingly, patients were classified into mild illness, pneumonia, and severe pneumonia (fever or suspected respiratory infection, plus one of the following: respiratory rate >30 breaths/min, severe respiratory distress, and SpO2 ≤ 93% on room air). Severe cases that needed oxygen therapy with no response to titrated oxygen therapy will require ICU treatment.

### Criteria for ICU Admission

All patients were managed by same protocol and were evaluated by the same intensivists available during their admission period. Admission criteria for ICU followed the hospital policy ABH/CLN/033/V02, 2017, revised 2020. All physicians followed the same protocol. Severity at the time of admission (as per the criteria already defined): Severe disease: 9/39, and Critical disease: 30/39.

### Blood and Radiological Tests

Laboratory tests were retrieved that include (1) complete blood count, including neutrophil count lymphocyte count, heamoglobin; Hb, white cell count; WCC, and platelets count, (2) coagulation profile, including interenational normalized ratio; INR, Prothrombin time; PT, (3) electrolytes, including sodium; Na and potassium; K, (4) renal function tests, including urea, creatinine, and estimated glomerular filtration rate; eGFR, (5) liver function tests, including total serum bilirubin, alanine aminotransferase; ALT, aspartate aminotransferase; AST, alkaline phosphatase; ALP, and albumin, (6) inflammatory markers, including C-reactive protein; CRP, D-dimers, lactate dehydrogenase; LDH, procalcitonin and ferritin. For risk of severe cases, the presence of lymphopenia, neutrophilia, high ALT/AST, high LDH, high CRP, high ferritin, high d-dimer, and high pro-calcitonin, above the age and gender-matched references were used as indicators of risk. Admission chest X-Ray (presence of bilateral air consolidation), and computerized tomography (CT) scan (presence of bilateral peripheral ground-glass opacities) were documented.

### Statistical Analysis

For all statistical analyses and tests, SPSS was used (Released 2019. IBM SPSS Statistics for Windows, Version 26.0. Armonk, NY: IBM Corp). The Chi-Square Test of Independence was used to examine the association between categorical variables while student's *t*-test was used for continuous variables groups means comparison.

Patients were grouped into those that didn't need ICU and those who needed it. ROC Curve, Precision-Recall Curve, and Overall model quality options in SPSS were used to identify predictors of ICU admission and their cutoff values.

The normality test for all groups was done by Shapiro-Wilk tests using SPSS, and sig. of all independent variables> 0.05 means that all groups were normally distributed.

## Results

### Patients Needed ICU Were Older Men With Less Contact History

Training cohorts (*n* = 128) were divided into those who needed ICU (*n* = 39, 30.5%) “ICU” and those who didn't need ICU (*n* = 89, 69.5%) “No ICU.” Patients who needed ICU were older (57 ± 13 years old) than No ICU patients (44 ± 15 years old) (*p* = 0.0001). 36 (92.3%) of the ICU group were males compared to 66 (74.2%) in the No ICU group (*p* = 0.019). Documented contact history with a positive case of COVID-19 was more prevalent in the No ICU group (*n* = 26, 29.2%) compared to (*n* = 3, 7.7%) in ICU patients, as shown in Table 1. There was no difference in their BMI (28.42–27.49) or Duration of illness before approaching the hospital (5–7 ± 3 days).

**Table 1:** Demographic characteristics of the training and validation cohort.

**Training cohort**		**ICU_Admin**						
		**No ICU**			**ICU**			***P*-value**
		**Count**	**Row *N* %**	**Column *N* %**	**Count**	**Row *N* %**	**Column *N* %**	
Sex	F	23	88.5	25.8	3	11.5	7.7	0.019^*^
	M	66	64.7	74.2	36	35.3	92.3	
Travel history	No	83	68.6	93.3	38	31.4	97.4	0.339^b^
	Yes	6	85.7	6.7	1	14.3	2.6	
Contact history	No	63	63.6	70.8	36	36.4	92.3	0.007^*^
	Yes	26	89.7	29.2	3	10.3	7.7	
**Validation cohort**		**ICU_Admin**						
		**No ICU**			**ICU**			***P*****-value**
		**Count**	**Row** ***N*** **%**	**Column** ***N*** **%**	**Count**	**Row** ***N*** **%**	**Column** ***N*** **%**	
Sex	F	70	89.7%	30.3%	8	10.3%	6.3%	0.000^*^
	M	161	57.5%	69.7%	119	42.5%	93.7%	
Travel history	No	211	62.8%	91.3%	125	37.2%	98.4%	0.008^*^
	Yes	20	90.9%	8.7%	2	9.1%	1.6%	
Contact history	No	165	59.6%	71.4%	112	40.4%	88.2%	0.000^*^
	Yes	66	81.5%	28.6%	15	18.5%	11.8%	

*Pearson Chi-Square Tests*.

* *The Chi-square statistic is significant at the 0.05 level*.

*N, number; F, females; M, males; ICU, intensive care unit; ICU_Admin, admission to the ICU*.

### Patients Needed ICU Presented More With SOB and Fever

Patients needed ICU admission presented more SOB (*n* = 29, 74.4%) than No ICU (*n* = 33, 37.1%), *P* < 0.001 and with fever (*n* = 30, 76.9%) compared to No ICU (*n* = 53, 59.6%), *p* = 0.05), as shown in Table 2.

**Table 2:** Chief presentation in the training cohort.

	**ICU_Admin**						
		**No ICU**			**ICU**			***P*-value**
		**Count**	**Row *N* %**	**Column *N* %**	**Count**	**Row *N* %**	**Column *N* %**	
SOB	No	56	84.8	62.9	10	15.2	25.6	0.000^*^
	Yes	33	53.2	37.1	29	46.8	74.4	
Fever	No	36	80.0	40.4	9	20.0	23.1	0.058
	Yes	53	63.9	59.6	30	36.1	76.9	
Cough	No	43	70.5	48.3	18	29.5	46.2	0.822
	Yes	46	68.7	51.7	21	31.3	53.8	
Fatigue	No	81	68.1	91.0	38	31.9	97.4	0.191
	Yes	8	88.9	9.0	1	11.1	2.6	
Anorexia	No	88	69.8	98.9	38	30.2	97.4	0.545
	Yes	1	50.0	1.1	1	50.0	2.6	
Sputum production	No	87	69.6	97.8	38	30.4	97.4	0.913
	Yes	2	66.7	2.2	1	33.3	2.6	
Myalgias	No	74	67.3	83.1	36	32.7	92.3	0.170
	Yes	15	83.3	16.9	3	16.7	7.7	
Headache	No	82	68.9	92.1	37	31.1	94.9	0.577
	Yes	7	77.8	7.9	2	22.2	5.1	
Rhinorrhea	No	85	69.1	95.5	38	30.9	97.4	0.604
	Yes	4	80.0	4.5	1	20.0	2.6	
Sore throat	No	82	68.9	92.1	37	31.1	94.9	0.577
	Yes	7	77.8	7.9	2	22.2	5.1	
Vomiting	No	88	69.3	98.9	39	30.7	100.0	0.506
	Yes	1	100.0	1.1	0	0.0	0.0	
Diarrhea	No	85	69.1	95.5	38	30.9	97.4	604
	Yes	4	80.0	4.5	1	20.0	2.6	
Nausea	No	87	69.0	97.8	39	31.0	100.0	0.345
	Yes	2	100.0	2.2	0	0.0	0.0	

*Pearson Chi-Square Tests*.

* *The Chi-square statistic is significant at the 0.05 level*.

*N, number; ICU, intensive care unit; ICU_Admin, admission to the ICU; SOB, shortness of breath*.

### Patients Needed ICU Showed Distinct Laboratory Findings

Patients needed ICU admission showed a higher Neutrophil count, WCC, Urea, creatinine, AST, CRP, D Dimer, LDH, Ferritin, and Troponin but lower Hb, eGFR, and albumin compared with No ICU patients, as shown in Table 3.

**Table 3:** Laboratory parameters in the training cohort.

	**ICU_Admin**				
	**No ICU**		**ICU**		***P*-value**
	**Mean**	**Standard error of mean**	**Mean**	**Standard error of mean**	
Neutrophil count ( × 10(3)/mcL)	5.44	0.38	8.46	0.74	*P <* 0.05
Hemoglobin (gm/dL)	13.56	0.21	12.58	0.27	*P <* 0.05
WBC (×10(3)/mcL)	7.69	0.39	9.93	0.83	*P <* 0.05
Urea (mmol/L)	4.90	0.26	11.60	2.53	*P <* 0.05
Creatinine (umol/L)	86.214	5.076	174.188	57.107	*P <* 0.05
eGFR (mL/min/1.73m^2^)	95.6	3.1	70.8	5.3	*P <* 0.05
AST (U/L)	44	3	61	6	*P <* 0.05
Albumin (gm/L)	33.1	0.7	25.8	1.1	*P <* 0.05
CRP (mg/l)	64.65	14.48	161.85	16.45	*P <* 0.05
D-dimers (mg/dL)	2	1	7	2	*P <* 0.05
LDH (IU/L)	346	20	530	41	*P <* 0.05
Ferritin (mcg/L)	933	137	1,773	295	*P <* 0.05
Troponin (ng/ml)	25	10	234	122	*P <* 0.05
ALP (IU/L)	87.56	5.26	105.49	10.17	ns
Lymphocyte count (×10(3)/mcL)	3.62	2.05	0.91	0.07	ns
Platelet count (×10(3)/mcL)	239	10	243	14	ns
INR (seconds)	1	0	1	0	ns
Prothrombin time (seconds)	13	0	13	0	ns
Na (mmol/L)	136.8	0.4	136.9	0.9	ns
K (mmol/L)	4.09	0.06	4.21	0.12	ns
S bil (μmol/L)	17.3	4.8	17.8	2.1	ns
ALT (IU/L)	62	5	58	6	ns
Procalcitonin (ug/L)	1	1	3	2	ns
Lactic acid	2	0	2	0	ns
Hba1c (%)	7.9	0.3	8.2	0.3	ns

*ICU, intensive care unit; ICU_Admin, admission to the ICU; p-value significant > 0.05, WCC, white cell count; eGFR, estimated glomerular filtration rate; AST, Aspartate aminotransferase; CRP, C-reactive protein; LDH, lactate dehydrogenase; ALP, Alkaline phosphatase; INR, international normalized ratio; Na, sodium; K, potassium; S bil, serum bilirubin; ALT, Alanine aminotransferase; HbA1C, glucosylated hemoglobin*.

*T-test was used to compare means between the groups*.

### Patients Needed ICU Showed More CXR and Less CT Scan Opacities

Patients needed ICU admission showed more frequency of consolidations identified by chest X-Ray (*n* = 32, 82.1%) than No ICU group (*n* = 43, 57.3%), *p* = 0.001. Bilateral peripheral ground-glass opacities (GGO) documented by chest CT scan was higher in No ICU patients (*n* = 64, 71.9%) compared to (*n* = 23, 59.0%) in ICU patients, as shown in Table 4.

**Table 4:** Chest X-Ray and CT Scan on admission in the training cohort.

		**ICU_Admin**						***P*-value**
		**No ICU**			**ICU**			
		**Count**	**Row *N* %**	**Column *N* %**	**Count**	**Row *N* %**	**Column *N* %**	
CXR	Consolidation	43	57.3	48.3	32	42.7	82.1	0.001^*^
	Normal	33	91.7	37.1	3	8.3	7.7	
	NOT Done	13	76.5	14.6	4	23.5	10.3	
CT chest:	Bilateral peripheral ground-glass opacities.	64	73.6	71.9	23	26.4	59.0	0.000^*^
	Normal	23	85.2	25.8	4	14.8	10.3	
	NOT Done	2	14.3	2.2	12	85.7	30.8	

*Pearson Chi-Square Tests*.

* *The Chi-square statistic is significant at the 0.05 level*.

*N, number; ICU, intensive care unit; ICU_Admin, admission to the ICU; CXR, chest X-ray; CT, computed tomography*.

### Patients Needed ICU Required Multiple In-patient Treatments

More patients needed ICU admission were treated with Lopinavir-ritonavir (*n* = 32, 82.1%); *p* = 0.05, Favipiravir (*n* = 19, 48.7%); *p* = 0.024, intravenous (IV) AB(*n* = 38, 97.4%); *P* < 0.001, IV steroids (*n* = 32, 100%); *P* < 0.001, Tocilizumab (*n* = 26, 66.7%); *P* < 0.001, and Antifungal (*n* = 10, 25.6%); *P* < 0.001 than No ICU patients, as shown in Table 5.

**Table 5:** Major medications used in the training cohort.

**In-patient treatment**		**ICU_Admin**						***P*-Value**
		**No ICU**			**ICU**			
		**Count**	**Row *N* %**	**Column *N* %**	**Count**	**Row *N* %**	**Column *N* %**	
Lopinavir-ritonavir	No	31	81.6	34.8	7	18.4	17.9	0.054
	Yes	58	64.4	65.2	32	35.6	82.1	
Favipiravir	No	64	76.2	71.9	20	23.8	51.3	0.024^*^
	Yes	25	56.8	28.1	19	43.2	48.7	
IV antibiotics	No	47	97.9	52.8	1	2.1	2.6	0.000^*^
	Yes	42	52.5	47.2	38	47.5	97.4	
IV steroids	No	57	89.1	64.0	7	10.9	17.9	0.000^*^
	Yes	32	50.0	36.0	32	50.0	82.1	
Tocilizumab	No	83	86.5	93.3	13	13.5	33.3	0.000^*^
	Yes	6	18.8	6.7	26	81.3	66.7	
Antifungal	No	84	74.3	94.4	29	25.7	74.4	0.000^*^
	Yes	5	33.3	5.6	10	66.7	25.6	
Chl/HQ	No	4	44.4	4.5	5	55.6	12.8	0.090
	Yes	85	71.4	95.5	34	28.6	87.2	
Interferon	No	67	72.8	75.3	25	27.2	64.1	0.195
	Yes	22	61.1	24.7	14	38.9	35.9	

*Pearson Chi-Square Tests*.

* *The Chi-square statistic is significant at the 0.05 level*.

*N, number; ICU, intenseive care unit; ICU_Admin, admission to the ICU; IV, intravenous; Chl/HQ, chloroquine/hydroxychloroquine*.

### Patients Needed ICU Showed a Higher Rate of Clinical Complications and Death

Patients needed ICU admission developed more complications like Acute cardiac injury, Acute kidney injury, acute liver injury, Acidosis, Ventilated, Acute Respiratory Distress Syndrome (ARDS), Septic shock with higher mortality when compared to No ICU patients, as shown in Table 6.

**Table 6:** In-hospital complication developed in the training cohort.

**Complications**		**ICU_Admin**						***P*-value**
	**No ICU**			**ICU**			
		**Count**	**Row *N* %**	**Column *N* %**	**Count**	**Row *N* %**	**Column *N* %**	
Acute cardiac injury	No	85	87.6	95.5	12	12.4	30.8	0.000^*^
	Yes	4	12.9	4.5	27	87.1	69.2	
Acute kidney injury	No	86	89.6	96.6	10	10.4	25.6	0.000^*^
	Yes	3	9.4	3.4	29	90.6	74.4	
Acute liver injury	No	81	78.6	91.0	22	21.4	56.4	0.000^*^
	Yes	8	32.0	9.0	17	68.0	43.6	
Acidosis	No	89	89.0	100.0	11	11.0	28.2	0.000^*^
	Yes	0	0.0	0.0	28	100.0	71.8	
Ventilated	No	88	88.0	98.9	12	12.0	30.8	0.000^*^
	Yes	1	3.6	1.1	27	96.4	69.2	
ARDS	No	82	94.3	92.1	5	5.7	12.8	0.000^*^
	Yes	7	17.1	7.9	34	82.9	87.2	
Septic shock	No	88	88.0	98.9	12	12.0	30.8	0.000^*^
	Yes	1	3.6	1.1	27	96.4	69.2	
Death	No	89	89.0	100.0	11	11.0	28.2	0.000^*^
	Yes	0	0.0	0.0	28	100.0	71.8	

*Pearson Chi-Square Tests*.

* *The Chi-square statistic is significant at the 0.05 level*.

*N, number; ICU, intensive care unit; ICU_Admin, admission to the ICU; ARDS, acute respiratory distress syndrome*.

### High D Dimer, Troponin, and Urea Can Positively Predict the Admission to ICU in Patients With COVID-19

**Figure 1:**
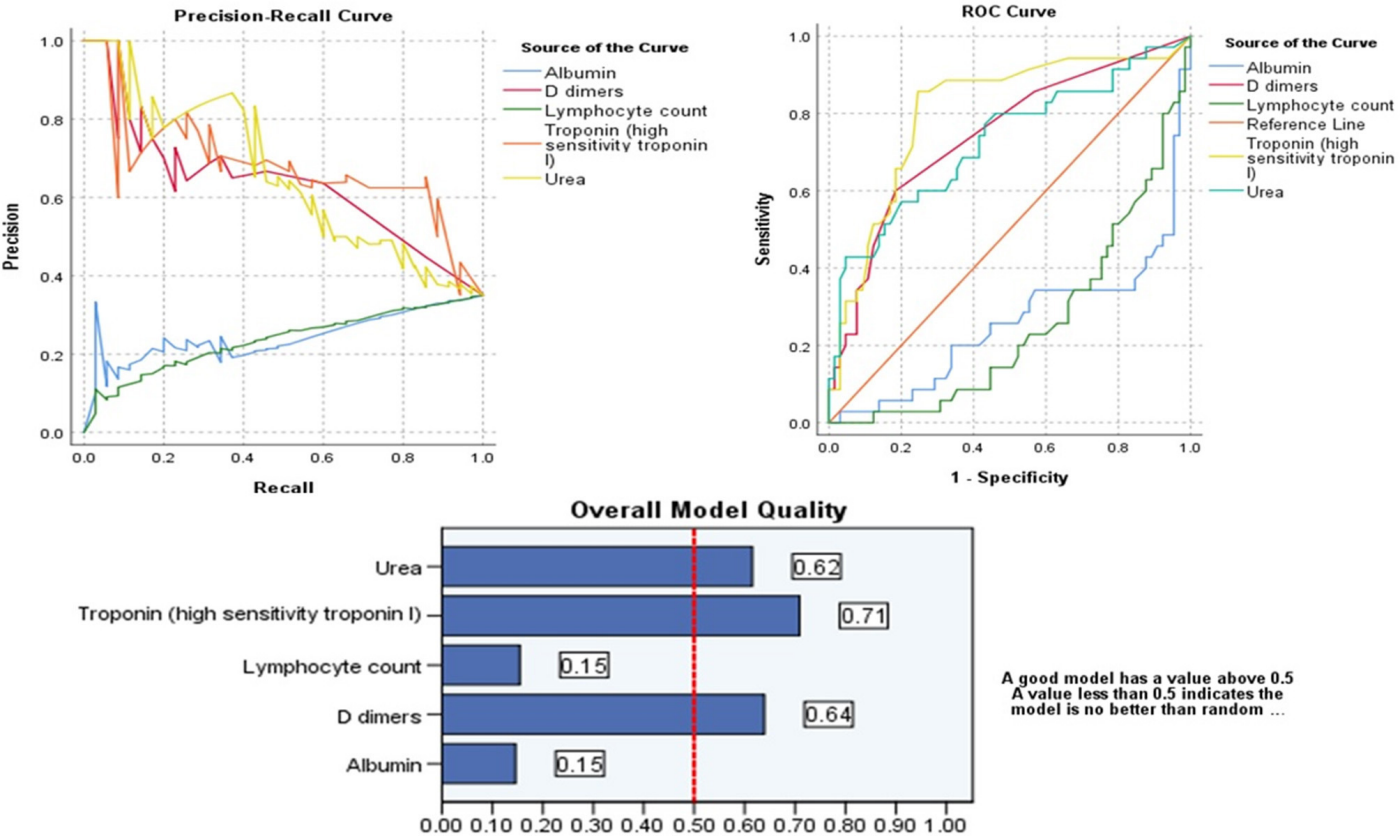
Probabilistic forecasts for binary classification (ICU vs. NO ICU) predictive modeling using ROC Curves, Precision-Recall curves of the significant parameters and Overall Model Quality.

Probabilistic forecasts for binary classification (ICU vs. NO ICU) predictive modeling using ROC Curves and Precision-Recall curves showed that among all variables D dimers (>1.5 mg/dl), Urea (>6.5 mmol/L), and Troponin >13.5 ng/ml) can positively predict the admission to ICU in patients with COVID-19. On the other hand, decreased Lymphocyte count and albumin can predict admission to ICU in patients with COVID-19, as shown in Table 7 and Figure 1.

**Table 7:** Probabilistic forecasts for binary classification (ICU vs. NO ICU) predictive modeling using ROC Curves and Precision-Recall curves.

**Area under the ROC curve**					
**Test result variable(s)**	**Area**	**Std. error**	**Asymptotic Sig**.	**Asymptotic 95% confidence interval**	
				**Lower bound**	**Upper bound**
Troponin	0.804	0.048	0.000	0.709	0.899
D dimers	0.744	0.053	0.000	0.639	0.848
Urea	0.726	0.056	0.000	0.616	0.836
Lymphocyte count	0.252	0.050	0.000	0.154	0.349
Albumin	0.256	0.056	0.000	0.146	0.366

*The test result variable(s): Troponin (high sensitivity troponin I) ND, not done; D, dimers; Urea, Lymphocyte count, albumin has at least one tie between the positive actual state group and the negative actual state group. Statistics may be biased+*.

### Validation of the Three Predictors on a Larger Cohort of COVID-19 Patients

In order to validate the performance of these three markers in a larger cohort, we explored the Sensitivity and Specificity of using these parameters in the 289-validation cohort who attended the hospital between March and June 2020. Patients who showed D dimers (>1.5), Urea (>6.5 mmol/L), and Troponin >13.5 ng/ml were checked whether they would need ICU admission or not. Using the three markers gave low sensitivity (30.25%; 22.17–39.35%) but high specificity (93.10%; 88.26–96.39%) to predict ICU admission. Using any two of the three markers gave moderate sensitivity (59.32%, 49.89–68.27%) but high specificity (79.31%; 72.53–85.07%) to predict ICU admission. Using any one of the three markers gave high sensitivity (85.47%; 77.76–91.30%) but low specificity (45.98%; 38.41–53.68%) to predict ICU admission, as shown in Table 8.

**Table 8:** Validation of the three predictors on a larger cohort (*n* = 289) of COVID-19 patients suing to show different Sensitivity, Specificity, Positive Likelihood Ratio, and Negative Likelihood Ratio.

	**Using the 3 markers**		**Using any 2 of the markers**		**Using any 1 of the markers**	
**Statistic**	**Value**	**95% CI**	**Value**	**95% CI**	**Value**	**95% CI**
Sensitivity	30.25	22.17–39.35	59.32	49.89–68.27	85.47	77.76–91.30
Specificity	93.10	88.26–96.39	79.31	72.53–85.07	45.98	38.41–53.68
Positive likelihood ratio	4.39	2.38–8.08	2.87	2.07–3.98	1.58	1.35–1.85
Negative likelihood ratio	0.75	0.66–0.85	0.51	0.41–0.65	0.32	0.20–0.50

## Discussion

Better understanding and identification of risk factors that might predispose for ICU admission might be essential for more active medical decision-making that might lead to optimal clinical practice to improve patients outcomes. Our results showed a significant difference in the pre-admission demographic, clinical, as well as laboratory characteristics of the ICU, admitted group when compared to non-admitted group.

Our study showed that patients needed ICU were older men with less contact history. Indeed, the association between ICU admission and elder age group was previously described in several reports that also showed the median age of critical/death groups to be higher than that of non-critical group groups (15–17). Older age and male sex were found to be independent risk factors for poor outcome of the illness in many reports (18). Specifically, older patients (>65 years) with comorbidities are at increased risk of death (19) where older age can independently predict the 60-day mortality after ICU admission (20). Such patients need careful observation and early intervention to prevent the potential development of severe COVID-19 (21).

On the other hand, disparities in the sex and gender observed in COVID-19 vulnerability was documented in many parts of the world (22). The majority of affected patients have been male who had more refractory disease and death (23). Gender differences in the COVID-19 outbreak should be taking into consideration in understanding the disease burden and dynamics of health emergency on individuals and communities (24). Male patients with heart injury, hyperglycemia, and high-dose corticosteroid were shown to have a high risk of death (21).

Our note that documented contact history with a positive case of COVID-19 was more prevalent in the No ICU group compared to ICU patients brings attention to the importance of the part of the asymptomatic carrier of the stories. Having contact with such asymptomatic carriers might be long enough to have more chances to present with the severe disease than the COVID-19 patients who came with known contact history as he will seek help earlier in the course of the disease. Asymptomatic carriers are prone to be mildly ill with the communicable period up to 3 weeks, and the communicated patients could develop severe illness (25).

Patients needed ICU admission presented more SOB and fever than No ICU patients. A systematic literature review and meta-analysis showed that among clinical manifestations, like fever, shortness of breath, or dyspnea, were associated with the progression of the disease (15). It is widely accepted that most of the COVID-19 patients will present with fever, cough, fatigue, and dyspnea, and fever is the most common symptom in patients with COVID-19 (26). Patients with severe COVID-19 are reported to have more silent hypoxemia, and coronavirus was suspected of having an idiosyncratic action on receptors involved in chemosensitivity to oxygen (27). Additionally, higher ferritin was higher in our cohort, which needed ICU admission compared to the No ICU group. Some reports document that high serum ferritin is a poor prognostic factor (28) as it represents a crucial mediator of immune dysregulation, contributing to the cytokine storm with fatal outcomes in COVID-19 (29).

Patients needed ICU admission showed a lower hemoglobin level compared with No ICU patients. So another explanation was linked to a hemoglobinopathy, hypoxia, and cell iron overload in COVID-19 patients due to direct SARS-CoV-2 interaction with hemoglobin molecule or hepcidin-mimetic action of a viral spike protein leading to an oxygen-deprived blood disease, with iron metabolism dysregulation (30). In a case study, recombinant human erythropoietin (rhEPO) was suggested to attenuate respiratory distress syndrome by enhancing leukocyte release from bone marrow and iron redistribution away from the intracellular virus (31).

Patients needed ICU admission showed a higher Neutrophil count and WCC compared with No ICU patients. Autopsy of COVID-19 patient's lungs showed infiltration of neutrophils with an aberrant neutrophil extracellular traps (NETs) formation that correlates with the clinical severity of COVID-19 (32). Enhanced neutrophil infiltration can induce necroinflammation that contributes to the higher mortality of COVID-19 in patients with underlying comorbidities (33). Recently, it was suggested that SARS-CoV2 could evade the innate immune response, causing uncontrolled NETs formation that leads to multi-organ failure (34).

Higher urea, creatine, and lower eGFR in ICU patients indicate the well-documented impact of COVID-19 on renal functions. Preexisting kidney disease on admission and/or the development of acute kidney injury (AKI) in patients with COVID-19 during hospitalization is high and is associated with in-hospital mortality (35). Usually, such AKI is resolved within 3 weeks after the onset of symptoms, but renal complications will lead specifically to higher mortality (36).

Patients needed ICU admission showed a higher AST and lowered albumin compared with No ICU patients that might indicate a hepatic injury. Digestive symptoms and liver injury have been reported during the course of the COVID-19 (37). It is well–known that patients with COVID-19 had liver comorbidities or reported abnormal levels of alanine aminotransferase and aspartate aminotransferase (AST) during disease progression (38). It is uncertain whether the COVID-19-related liver damage/dysfunction is caused by direct viral infection, as a consequence of the use of potentially hepatotoxic drugs, or as part of the multiple organ dysfunction in COVID-19 (39).

Patients needed ICU admission showed a higher C-reactive protein (CRP) and LDH compared with No ICU patients. This goes with the reports that found that the level of plasma CRP was positively correlated to the severity of COVID-19 pneumonia and can be useful as an earlier indicator for severe illness (40). In severe COVID-19 patients, CRP increased significantly before CT findings. Importantly, CRP, which was associated with disease development (41). Patients with positive RT-PCR had a significantly higher neutrophil count, CRP, lactate dehydrogenase (LDH), and Urea levels in serum (42). Serum LDH decline was shown to predict a favorable response to the treatment of COVID-19 infection (43).

Patients needed ICU admission showed more frequency of consolidations identified by chest X-Ray than the No ICU group. Bilateral peripheral GGO documented by chest CT scan was higher in No ICU patients compared to ICU patients. Chest CT is well-accepted as a standard method for the rapid diagnosis of COVID-19 (44). Typically COVID-19 pneumonia CT imaging abnormalities vary from focal unilateral to diffuse bilateral GGO that can be detected even in asymptomatic patients (45). Identification of GGO and a single lesion on the initial CT scan might suggest early-phase disease (46). So the lower incidence of GGO in the ICU group might indicate either it is not specific to severe cases or might indicate a false indication that the cases are not severe and might delay the actions needed.

Our results showed that patients who showed D dimers (>1.5 mg/dl), Urea (>6.5 mmol/L), and Troponin >13.5 ng/ml will have a higher chance of developing critical COVID-19 and will need ICU admission with higher complications and mortality. Using any two of the three markers gave moderate sensitivity (59.32%; 49.89–68.27%) but high specificity (79.31%; 72.53–85.07%) to predict ICU admission. High serum levels of D-Dimers and LDH in the absence of anticoagulation were associated with 1-month mortality among older inpatients with Covid-19 (47). Du et al. recently identified age ≥65 years, and cardiac troponin I ≥ 0.05 ng·mL-1 as two of four risk factors and predictors for mortality of COVID-19 pneumonia patients (48).

Using those bedside tests that can be done even outside the emergency department can save lives and resources by directing the service provider about the group of patients that might deteriorate and need admission to ICU. Directing such patients earlier to hospital with ICU might decrease the mortality and control the spread.

## Conclusion

This demonstrated the accuracy of our approach in identifying factors that can predict the COVID 19 patient outcome. For that reason, the stratification of patients according to the parameters discovered by our model might provide a simple and efficient system for patients' risk stratification. This system might help clinicians and health care providers to deliver more efficient medical care for COVID 19 patients. Those factors, in addition to the fact that this report is the first report that uses in house patient cohort from UAE highlight the importance of implementation of such a method in the stratification of our own patients into high or low-risk groups for ICU transfer might be essential for more efficient use of our own resources and infrastructures available to deal with the COVID 19 outbreak.

## Data Availability Statement

The original contributions presented in the study are included in the article/supplementary materials, further inquiries can be directed to the corresponding author/s.

## Ethics Statement

The studies involving human participants were reviewed and approved by the Scientific Research Committee MOHAP/DXB-REC/MMM/NO.44/2020 and certify that the study was performed in accordance with the ethical standards as laid down in the 1964 Declaration of Helsinki and its later amendments ethical standards. Written informed consent for participation was not required for this study in accordance with the national legislation and the institutional requirements.

## Author Contributions

All authors listed have made a substantial, direct and intellectual contribution to the work, and approved it for publication.

## Conflict of Interest

The authors declare that the research was conducted in the absence of any commercial or financial relationships that could be construed as a potential conflict of interest.

## References

[1] Wenham C Smith J Morgan R Gender Group C-W. COVID-19: the gendered impacts of the outbreak. Lancet. (2020) 395:846–8. 10.1016/S0140-6736(20)30526-2 32151325 PMC7124625

[2] Wu Z McGoogan JM. Characteristics of and important lessons from the coronavirus disease 2019. (COVID-19) outbreak in china: summary of a report of 72314 cases from the chinese center for disease control and prevention. JAMA. (2020) 323:1239–42. 10.1001/jama.2020.2648 32091533

[3] Jin JM Bai P He W Wu F Liu XF Han DM. Gender differences in patients with covid-19: focus on severity and mortality. Front Public Health. (2020) 8:152. 10.3389/fpubh.2020.00152 32411652 PMC7201103

[4] Grasselli G Pesenti A Cecconi M. Critical care utilization for the covid-19 outbreak in lombardy, italy: early experience and forecast during an emergency response. JAMA. (2020) 323:1545–6. 10.1001/jama.2020.4031 32167538

[5] Allenbach Y Saadoun D Maalouf G Vieira M Hellio A Boddaert J. Multivariable prediction model of intensive care unit transfer and death: a French prospective cohort study of COVID-19 patients. PLoS ONE. medRxiv. [Preprint]. (2020). 10.1101/2020.05.04.20090118 33075088 PMC7571674

[6] Arabi YM Murthy S Webb S COVID-19: a novel coronavirus and a novel challenge for critical care. Intensive Care Med. (2020) 46:833–6. 10.1007/s00134-020-05955-1 32125458 PMC7080134

[7] Maves RC Downar J Dichter JR Hick JL Devereaux A Geiling JA. Triage of scarce critical care resources in COVID-19 an implementation guide for regional allocation: an expert panel report of the task force for mass critical care and the American college of chest physicians. Chest. (2020) 158:212–25. 10.1016/j.chest.2020.03.063 32289312 PMC7151463

[8] Einav S Hick JL Hanfling D Erstad BL Toner ES Branson RD Surge capacity logistics: care of the critically ill and injured during pandemics and disasters: CHEST consensus statement. Chest. (2014) 146(4 Suppl):e17S−43S. 10.1378/chest.14-0734 25144407

[9] Gong J Ou J Qiu X Jie Y Chen Y Yuan L. A tool to early predict severe 2019-novel coronavirus pneumonia (covid-19): a multicenter study using the risk nomogram in Wuhan and Guangdong, China. medRxiv. [Preprint]. (2020). 10.1101/2020.03.17.20037515 32296824 PMC7184338

[10] Wynants L Van Calster B Bonten MMJ Collins GS Debray TPA De Vos M. Prediction models for diagnosis and prognosis of covid-19 infection: systematic review and critical appraisal. BMJ. (2020) 369:m1328. 10.1101/2020.03.24.20041020 32265220 PMC7222643

[11] Ali I. COVID-19: are we ready for the second wave? Disaster Med Public Health Prep. (2020) 2020:1–3. 10.1017/dmp.2020.149 32379015 PMC7239772

[12] Jiang X Coffee M Bari A Wang J Jiang X Huang J Towards an artificial intelligence framework for data-driven prediction of coronavirus clinical severity. Comput Mater Continua. (2020) 63:537–51. 10.32604/cmc.2020.010691

[13] Shang Y Liu T Wei Y Li J Shao L Liu M. Scoring systems for predicting mortality for severe patients with COVID-19. EClinicalMedicine. (2020) 24:100426. 10.1016/j.eclinm.2020.100426 32766541 PMC7332889

[14] Bartoletti M Giannella M Scudeller L Tedeschi S Rinaldi M Bussini L. Development and validation of a prediction model for severe respiratory failure in hospitalized patients with SARS-CoV-2 infection: a multicentre cohort study (PREDI-CO study). Clin Microbiol Infect. (2020) 26:1545–53. 10.1016/j.cmi.2020.08.003 32781244 PMC7414420

[15] Zheng Z Peng F Xu B Zhao J Liu H Peng J. Risk factors of critical & mortal COVID-19 cases: a systematic literature review and meta-analysis. J Infect. (2020) 81:e16–25. 10.1016/j.jinf.2020.04.021 32335169 PMC7177098

[16] Peng YD Meng K Guan HQ Leng L Zhu RR Wang BY. Clinical characteristics and outcomes of 112 cardiovascular disease patients infected by 2019-nCoV. Zhonghua Xin Xue Guan Bing Za Zhi. (2020) 48:E004. 10.3760/cma.j.cn112148-20200220-00105 32120458

[17] Tian S Hu N Lou J Chen K Kang X Xiang Z. Characteristics of COVID-19 infection in Beijing. J Infect. (2020) 80:401–6. 10.1016/j.jinf.2020.02.018 32112886 PMC7102527

[18] Wang D Yin Y Hu C Liu X Zhang X Zhou S. Clinical course and outcome of 107 patients infected with the novel coronavirus, SARS-CoV-2, discharged from two hospitals in Wuhan, China. Crit Care. (2020) 24:188. 10.1186/s13054-020-02895-6 32354360 PMC7192564

[19] Yang X Yu Y Xu J Shu H Xia J Liu H. Clinical course and outcomes of critically ill patients with SARS-CoV-2 pneumonia in Wuhan, China: a single-centered, retrospective, observational study. Lancet Respir Med. (2020) 8:475–81. 10.1016/S2213-2600(20)30079-5 32105632 PMC7102538

[20] Xu J Yang X Yang L Zou X Wang Y Wu Y. Clinical course and predictors of 60-day mortality in 239 critically ill patients with COVID-19: a multicenter retrospective study from Wuhan, China. Crit Care. (2020) 24:394. 10.1186/s13054-020-03098-9 32631393 PMC7336107

[21] Li X Xu S Yu M Wang K Tao Y Zhou Y. Risk factors for severity and mortality in adult COVID-19 inpatients in Wuhan. J Allergy Clin Immunol. (2020) 146:110–8. 10.1016/j.jaci.2020.04.006 32294485 PMC7152876

[22] Gebhard C Regitz-Zagrosek V Neuhauser HK Morgan R Klein SL. Impact of sex and gender on COVID-19 outcomes in Europe. Biol Sex Differ. (2020) 11:29. 10.1186/s13293-020-00304-9 32450906 PMC7247289

[23] Walter LA McGregor AJ. Sex- and Gender-specific Observations and Implications for COVID-19. West J Emerg Med. (2020) 21:507–9. 10.5811/westjem.2020.4.47536 32302282 PMC7234726

[24] Ambrosino I Barbagelata E Ortona E Ruggieri A Massiah G Giannico OV. Gender differences in patients with COVID-19: a narrative review. Monaldi Arch Chest Dis. (2020) 90:318–24. 10.4081/monaldi.2020.1389 32449614

[25] Hu Z Song C Xu C Jin G Chen Y Xu X. Clinical characteristics of 24 asymptomatic infections with COVID-19 screened among close contacts in Nanjing, China. Sci China Life Sci. (2020) 63:706–11. 10.1007/s11427-020-1661-4 32146694 PMC7088568

[26] Hu Y Sun J Dai Z Deng H Li X Huang Q. Prevalence and severity of corona virus disease. 2019 (COVID-19): a systematic review and meta-analysis. J Clin Virol. (2020). 127:104371. 10.1016/j.jcv.2020.104371 32315817 PMC7195434

[27] Tobin MJ Laghi F Jubran A. Why COVID-19 silent hypoxemia is baffling to physicians. Am J Respir Crit Care Med. (2020) 202:356–60. 10.1164/rccm.202006-2157CP 32539537 PMC7397783

[28] Terpos E Ntanasis-Stathopoulos I Elalamy I Kastritis E Sergentanis TN Politou M. Hematological findings and complications of COVID-19. Am J Hematol. (2020) 95:834–47. 10.1002/ajh.25829 32282949 PMC7262337

[29] Vargas-Vargas M Cortés-Rojo C. Ferritin levels and COVID-19. Rev Panam Salud Publica. (2020) 44:e72. 10.26633/RPSP.2020.72 32547616 PMC7286435

[30] Cavezzi A Troiani E Corrao S. COVID-19: hemoglobin, iron, and hypoxia beyond inflammation. a narrative review. Clin Pract. (2020) 10:1271. 10.4081/cp.2020.1271 32509258 PMC7267810

[31] Hadadi A Mortezazadeh M Kolahdouzan K Alavian G. Does recombinant human erythropoietin administration in critically ill COVID-19 patients have miraculous therapeutic effects? J Med Virol. (2020) 92:915–8. 10.1002/jmv.25839 32270515 PMC7262240

[32] Barnes BJ Adrover JM Baxter-Stoltzfus A Borczuk A Cools-Lartigue J Crawford JM. Targeting potential drivers of COVID-19: Neutrophil extracellular traps. J Exp Med. (2020) 217:e20200652. 10.1084/jem.20200652 32302401 PMC7161085

[33] Tomar B Anders HJ Desai J Mulay SR. Neutrophils and neutrophil extracellular traps drive necroinflammation in COVID-19. Cells. (2020) 9:1383. 10.3390/cells9061383 32498376 PMC7348784

[34] Thierry AR Roch B. SARS-CoV2 may evade innate immune response, causing uncontrolled neutrophil extracellular traps formation and multi-organ failure. Clin Sci. (2020) 134:1295–300. 10.1042/CS20200531 32543703

[35] Cheng Y Luo R Wang K Zhang M Wang Z Dong L. Kidney disease is associated with in-hospital death of patients with COVID-19. Kidney Int. (2020) 97:829–38. 10.1016/j.kint.2020.03.005 32247631 PMC7110296

[36] Pei G Zhang Z Peng J Liu L Zhang C Yu C. Renal involvement and early prognosis in patients with COVID-19 pneumonia. J Am Soc Nephrol. (2020) 31:1157–65. 10.1681/ASN.2020030276 32345702 PMC7269350

[37] Lee IC Huo TI Huang YH. Gastrointestinal and liver manifestations in patients with COVID-19. J Chin Med Assoc. (2020) 83:521–3. 10.1097/JCMA.0000000000000319 32243269 PMC7176263

[38] Zhang C Shi L Wang F-S. Liver injury in COVID-19: management and challenges. Lancet Gastroenterol Hepatol. (2020) 5:428–30. 10.1016/S2468-1253(20)30057-1 32145190 PMC7129165

[39] Feng G Zheng KI Yan QQ Rios RS Targher G Byrne CD. COVID-19 and liver dysfunction: current insights and emergent therapeutic strategies. J Clin Transl Hepatol. (2020) 8:18–24. 10.14218/JCTH.2020.00018 32274342 PMC7132016

[40] Chen W Zheng KI Liu S Yan Z Xu C Qiao Z. Plasma CRP level is positively associated with the severity of COVID-19. Ann Clin Microbiol Antimicrob. (2020) 19:18. 10.1186/s12941-020-00362-2 32414383 PMC7227180

[41] Tan C Huang Y Shi F Tan K Ma Q Chen Y. C-reactive protein correlates with computed tomographic findings and predicts severe COVID-19 early. J Med Virol. (2020) 92:856–862. 10.1002/jmv.25871 32281668 PMC7262341

[42] Mardani R Ahmadi Vasmehjani A Zali F Gholami A Mousavi Nasab SD Kaghazian H. Laboratory parameters in detection of covid-19 patients with positive rt-pcr; a diagnostic accuracy study. Arch Acad Emerg Med. (2020) 8:e43. 32259132 PMC7130449

[43] Yuan J Zou R Zeng L Kou S Lan J Li X. The correlation between viral clearance and biochemical outcomes of 94 COVID-19 infected discharged patients. Inflamm Res. (2020) 69:599–606. 10.1007/s00011-020-01342-0 32227274 PMC7103893

[44] Li Y Xia L. Coronavirus Disease (2019). (COVID-19): role of chest CT in DIAGNOSIS AND MANAGEMENT. AJR Am J Roentgenol. (2020) 214:1280–6. 10.2214/AJR.20.22954 32130038

[45] Shi H Han X Jiang N Cao Y Alwalid O Gu J. Radiological findings from 81 patients with COVID-19 pneumonia in Wuhan, China: a descriptive study. Lancet Infect Dis. (2020) 20:425–34. 10.1016/S1473-3099(20)30086-4 32105637 PMC7159053

[46] Zhou S Wang Y Zhu T Xia L. CT features of coronavirus disease 2019. (COVID-19) pneumonia in 62 patients in Wuhan, China. AJR Am J Roentgenol. (2020) 214:1287–94. 10.2214/AJR.20.22975 32134681

[47] Bousquet G Falgarone G Deutsch D Derolez S Lopez-Sublet M Goudot FX. ADL-dependency, D-Dimers, LDH and absence of anticoagulation are independently associated with one-month mortality in older inpatients with Covid-19. Aging. (2020) 12:11306–13. 10.18632/aging.103583 32576712 PMC7343508

[48] Du RH Liang LR Yang CQ Wang W Cao TZ Li M. Predictors of mortality for patients with COVID-19 pneumonia caused by SARS-CoV-2: a prospective cohort study. Eur Respir J. (2020) 55:2000524. 10.1183/13993003.00524-2020 32269088 PMC7144257

[49] Hachim M Hachim IY Naeem KB Hannawi H Salmi IA Hannawi S Corona virus disease 2019. (COVID-19): intensive care admission prediction model. Res Square. [Preprint]. (2020). 10.21203/rs.3.rs-35442/v1

